# Effect of Different CAD/CAM Milling and 3D Printing Digital Fabrication Techniques on the Accuracy of PMMA Working Models and Vertical Marginal Fit of PMMA Provisional Dental Prosthesis: An In Vitro Study

**DOI:** 10.3390/polym14071285

**Published:** 2022-03-22

**Authors:** Marina Sidhom, Hanaa Zaghloul, Ihab El-Sayed Mosleh, Elzahraa Eldwakhly

**Affiliations:** 1 Fixed Prosthodontics Department, Faculty of Oral and Dental Medicine, Misr International University, Cairo 12677, Egypt; marina.fayek@miuegypt.edu.eg; 2 Conservative Dentistry, Faculty of Oral & Dental Medicine, Misr International University, Cairo 12677, Egypt; hanaa.zaghloul@miuegypt.edu.eg; 3 Fixed Prosthodontics, Faculty of Dentistry, King Abdulaziz University, Jeddah 22252, Saudi Arabia; ihab_mosleh_mostafa@yahoo.com; 4 Fixed Prosthodontics, Faculty of Dentistry, Cairo University, Cairo 11553, Egypt; 5 Department of Clinical Dental Sciences, College of Dentistry, Princess Nourah bint Abdulrahman University, P.O. Box 84428, Riyadh 11671, Saudi Arabia

**Keywords:** working dental model, tooth-supported FDPs, digital techniques, milling method, three-dimensional printing method, marginal fit

## Abstract

***Background:*** Minimal evidence exists on the efficacy of different digital manufacturing techniques in the fabrication of precise dental working models and provisional prosthesis. ***Aim of study:*** The objective was to evaluate the effect of two digital fabrication techniques (CAD/CAM milling and 3D printing) on the accuracy of PMMA working models and marginal fit of PMMA provisional prosthesis. ***Materials and methods:*** Two abutment teeth of modified typodont were prepared. A reference stone model was fabricated, and an optical impression was performed to obtain a CAD reference model. Four CAM milled working models and four printed working models were fabricated. CAD software was used to design the provisional prostheses. Group A tested four milled provisional prosthesis, and group B tested four 3D printed prosthesis. The 3D accuracy of working models was assessed by superimposition of the control reference working model on the CAD test working model. A stereo-optical microscope was used to assess vertical marginal fit of the provisional dental prosthesis. Student’s *t* and Mann–Whitney U tests were utilized to compare the two groups. ***Results:*** Results showed no statistically significant difference between the two tested groups. ***Conclusion:*** The two digital working model fabrication techniques recorded comparable accuracy. Similarly, 3D printed provisional prosthesis showed comparable marginal fit to the CAD/CAM milled ones.

## 1. Introduction

Provisional restorations are crucial for the success of the prosthetic treatment plan. A precisely adapted and well-finished provisional restoration has many functions, including pulp protection, positional stability of the abutments, and restoration of function and aesthetics. Moreover, they play a critical clinical role in oral rehabilitation cases, as they provide a prospective simulation of the final restoration. They present a valuable tool in reorganizing the occlusal scheme in cases with loss of vertical dimension and complicated oral rehabilitation cases. Provisionalization plays a significant role in evaluating aesthetics and phonetics during the treatment duration for perfecting the definitive restoration. Furthermore, it is key to successful periodontal management in compromised aesthetic cases [1,2,3,4].

Various techniques of provisional restoration fabrication have been employed and improved over time, starting from the different conventional techniques using resins or composites to digital CAD/CAM fabrication methods [5]. The conventional fabrication techniques of the provisional restorations have many drawbacks related to the materials’ properties and the employment technique [6,7]. Consequently, digital techniques were introduced to overcome these drawbacks [2]. Digital CAD/CAM manufacturing of provisional restorations can be subdivided into subtractive manufacturing techniques by milling and the additive manufacturing technique by 3D printing [8,9,10,11,12,13].

Various factors can affect the accuracy and marginal adaptation of the fixed dental prosthesis, including the accuracy of the final impression, the master cast fabrication, and the prosthesis fabrication procedures [14]. The final impression is the foundation for an accurate master cast and a well-fitting restoration [14,15]. Many researchers investigated the difference in accuracy between conventional physical impressions and optical impressions for a properly fitting restoration [16]. Studies [17,18] showed that optical impressions are more time-efficient and lead to higher operator and clinician satisfaction. They also concluded that optical impressions could produce acceptable FDPs with marginal gap of less than 120 µm, especially for single units and short FDPs [18].

It may seem that with the evolution of CAD/CAM technology, physical working models are not essential. However, physical casts, even with digital manufacturing techniques, allow for proper contouring, finishing, and polishing of the restorations, especially in complex oral rehabilitation cases. They also enable the evaluation of the marginal fit, occlusion, and proximal contacts. Working models are mandatory for the fabrication of manually veneered FDPs that are indicated for cases with challenging aesthetic demands. Therefore, all analog, digital, and partial digital workflows still require physical working models [19].

Fabrication of an accurate working model reduces the possibility of framework misfit that significantly affects the prognosis of the final prosthesis [20]. Hard dental stone models poured from conventional impressions are considered the gold standard for constructing dental models [21]. However, the conventional workflow for obtaining stone models is time-consuming, labor-intensive, and may incorporate many errors as a result of the multiple clinical and laboratory procedures needed [19,22,23].

Moreover, recently, after the evolution of the CAD/CAM system, optical impressions have been used instead of physical ones, and hence new fabrication techniques of the working models were required. There are thus currently different fabrication methods for dental models other than the poured stone casts, including milling and 3D printing [8,24,25,26].

CAD/CAM milling/machining technology is a digital technique in which different complex shapes, crowns, frameworks, or working models are fabricated by grinding resin blocks to achieve the desired geometry, designed by the CAD software [10]. It allows for producing precise, complex shapes as the working dental models. However, this technique has many disadvantages, such as the considerable waste of raw material and the accuracy of the dental models produced being dependent on the size of the cutting burs of the milling machine. Frequent wear of the milling burs also occurs during the production of large objects as the dental models [8,27].

Therefore, 3D printing technologies were introduced to this field to overcome some of the drawbacks of the subtractive technique. These methods gained popularity as additive techniques (layer upon layer). Moreover, they can be utilized to manufacture precise prostheses and models with minimal material waste and are considered economical and fast techniques. The techniques available for the fabrication of the working models include stereolithography (SLA), digital light processing (DLP), and material jetting (Multijet and Polyjet). The earliest and most commonly used printing technique is SLA, which utilizes ultraviolet (UV) scanning laser to sequentially cure liquid photopolymer resin layers [21,28]. The advantages of 3D printing techniques include reducing time and standardized production. This approach allows for the large-scale production of models with high-wear resistance. Fine details such as undercuts and complex internal geometries can be reproduced [19].

As previously mentioned, these recent fabrication techniques are also considered crucial factors affecting the accuracy of provisional restorations. The milling technique provides frameworks of higher consistency and precision than conventional techniques, as the resin blocks are cured under optimal conditions. It also saves time and effort by decreasing the patient’s discomfort [9,12]. However, this technique has some disadvantages, as mentioned earlier [5,8].

However, 3D printing technologies can produce finer details (undercuts and better anatomy) [1,8,12,29] compared to milling with the least material waste.

The long-term success of tooth-supported restorations depends on the accuracy and fit of the framework of the FDPs over the prepared abutments [14,15]. The marginal misfits can be categorized into vertical, horizontal, and absolute marginal misfits. The vertical marginal misfit measured parallel to the path of withdrawal of the framework is called the vertical marginal discrepancy. The horizontal marginal misfit measured perpendicular to the path of withdrawal of the framework is called the horizontal marginal discrepancy [30,31]. However, the absolute marginal discrepancy is the angular combination of marginal gap and extension error. Vertical and horizontal marginal discrepancies are measured perpendicularly from the framework or the internal surface of the margin of the crown to the outer edge of the finish line of the tooth [30].

McLean and von Fraunhofer [16] reported that the clinically accepted boundary value of the vertical marginal gap is considered to be ≤100−120 μm after a 5-year clinical study of 1000 restorations. Christenson [32] suggested a clinical goal of 25 to 40 μm for the marginal adaptation of cemented restorations. For CAD/CAM restorations, the generally acceptable marginal gap discrepancies are between 50 and 100 μm [33]. However, marginal discrepancies in the restoration can expose the luting cement to the oral environment, leading to increased cement dissolution and permitting the percolation of food and microorganisms, leading to gingival irritation, periodontal diseases, and secondary caries [4].

According to the literature review, many studies [1,29,34,35,36] compared the fit of the milled or the three-dimensional printed restorations and the conventional ones. Many studies [24,37,38,39] also compared the accuracy of the three-dimensional printed dental models and the conventional ones. However, there is a lack of knowledge regarding comparing three-dimensional printed and milled models and the effect of these different manufacturing techniques on the marginal fit of provisional and final fixed dental prostheses. Therefore, this study aimed to compare the dimensional accuracy of working models fabricated by two different digital methods, milling and three-dimensional printing, and to evaluate the effect of the two methods on the vertical marginal fit of tooth-supported provisional dental prosthesis. Therefore, the first null hypothesis of this study was that there is no difference in the three-dimensional accuracy of working models fabricated with the two digital methods (three-dimensional printing and milling). The second was that there is no difference in the vertical marginal fit between three-dimensionally printed and CAD/CAM milled tooth-supported provisional dental prosthesis.

## 2. Materials and Methods

### 2.1. Fabrication of the Reference Model

Due to the material-based in vitro nature of the study and that it did not include any human or animal subjects or tissues, the study was exempted from the institutional review board ethical approval.

A dentate typodont (El Banna, Cairo, Egypt) was modified by removing the mandibular left first molar to simulate a clinical situation of a partially edentulous arch to be restored with a three-unit tooth-supported FDP. The mandibular left second premolar and first molar teeth were prepared to receive a full coverage ceramic three-unit FDP. The amount of preparation was calibrated by midsagittal and buccal indices. A PVS (Edge PVS, MDC Dental, Zapopan, Jalisco, México) physical final impression was taken to fabricate the reference model [24,27,37]. It was then poured with a low expansion stone type IV (GC Fuji Rock EP, GC Europe N.V.Leuven, Belgium). It was used as a control master reference model [24].

### 2.2. Exporting the Standard Tessellation Language (STL) File

**Figure 1:**
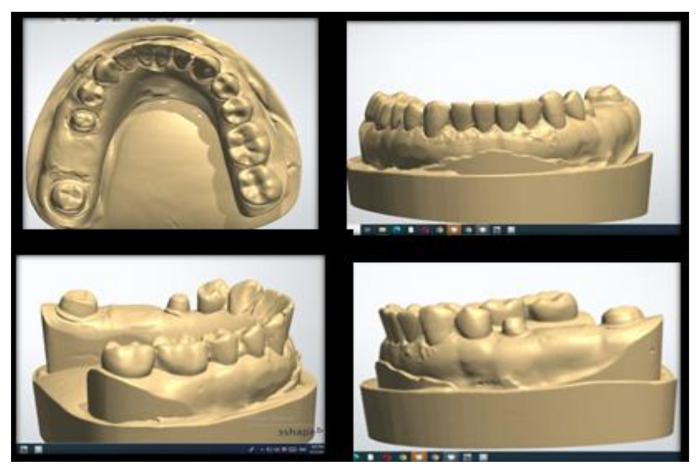
STL scan of the control reference model.

Digital impressions were taken by TRIOS 3 basic (3Shape, Copenhagen, Denmark) to mimic the clinical setting. Following the manufacturer’s recommendations, scanning of the reference model and opposing model and a buccal scan of the interarch relationship were carried out. Scans were exported to obtain the final virtual 3D master model as shown in Figure 1, and they were used to fabricate the test models and design the provisional dental prosthesis using CAD/CAM technology [24,40].

### 2.3. Sample Size Calculation

This power analysis used accuracy as the primary outcome. The effect size (d = 2.6798) was calculated based upon the results of Jeong YG et al. (2018) [8]. Using an alpha (α) level of (5%) and beta (β) level of (20%), i.e., power = 80%, the minimum estimated sample size was 4 specimens per group giving a total of 8 specimens. Sample size calculation was performed using G*Power Version 3.1.9.2.

### 2.4. Sample Grouping

For the working models, eight working models were classified into two groups (n = 4) according to the method of construction: group A, manufactured by the milling technique, and group B, manufactured by the three-dimensional printing technique.

For the tooth-supported provisional dental prosthesis grouping, eight tooth-supported provisional dental prostheses were classified into two groups (n = 4) according to the method of construction for measuring the marginal fit of provisional dental prosthesis: group A, manufactured by the milling technique, and group B, manufactured by the three-dimensional printing technique.

### 2.5. Fabrication of the Test Working Models

#### 2.5.1. Trimming of the Optical Scan Data

The model builder module of the dental CAD 3Shape software (3Shape, Copenhagen, Denmark) was used to adjust the STL file of the reference model scan before the milling and printing of the test groups. The scans were trimmed to remove the erroneous parts of scan data and select only the portion of the scan of concern to be milled or printed. A hollow 3D model was designed for the printed test groups and a solid model for the milled test groups. Finally, the digital model was exported in STL file format.

#### 2.5.2. Milling of the Test Working Models

**Figure 2:**
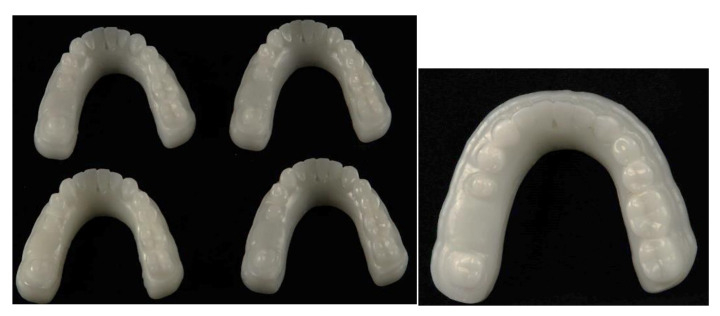
Milled working model.

Working models of group A were milled (n = 4) from the trimmed STL file using a PMMA disk (CAD-Ivory, On dent, Bornova, Turkey) as shown in Figure 2. The working models were milled following the manufacturer’s instructions using a five-axis milling machine (CAM 5-S1 impression milling machine, Vhf, Baden-Württemberg, Germany). The virtual model imported to the milling machine software was positioned on the blank, and the milling order was started.

#### 2.5.3. Printing of the Test Working Models

**Figure 3:**
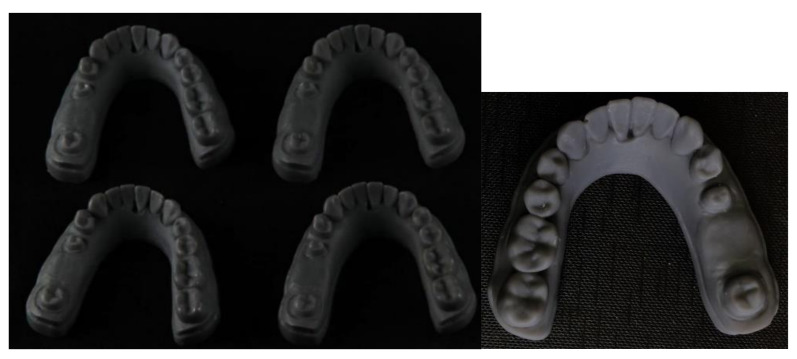
Printed working model.

Working models of group B (n = 4) were printed following the manufacturer’s instructions using a stereolithography (SLA) 3D printer (Formlabs Inc., Somerville, MA, USA), as shown in Figure 3. Hollow models were oriented directly on the built platform. The file was uploaded to the printer. The printed models were rinsed with isopropyl alcohol (IPA, 90%) until the uncured resin was thoroughly cleaned with the aid of the form wash unit (Formlabs Inc., Somerville, MA, USA). For optimal mechanical properties, accuracy, and precision, the models were post-cured by exposure to light and heat using the form cure unit (Formlabs Inc., Somerville, MA, USA).

### 2.6. Designing and Fabricating the Tooth-Supported Provisional Dental Prosthesis

#### 2.6.1. Restoration Design

**Figure 4:**
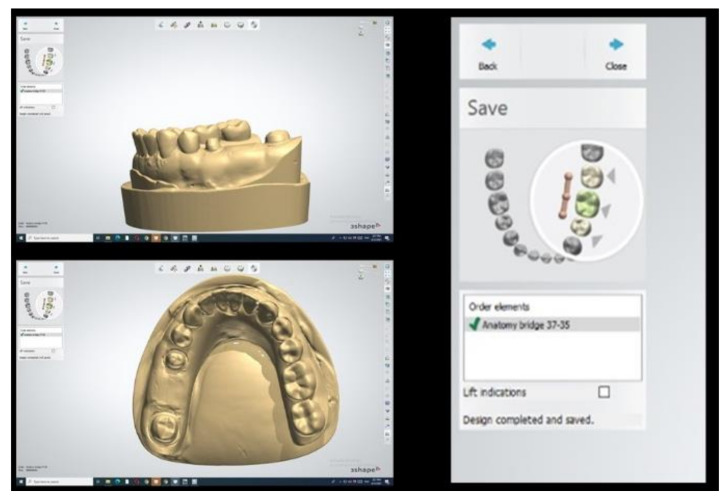
Selection of design.

**Figure 5:**
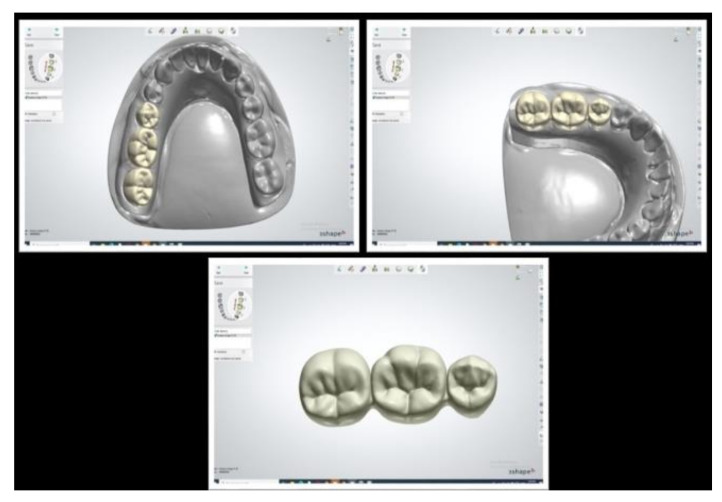
Finished FDP design. Milling the tooth-supported provisional dental prosthesis (group A).

Interim FDP was designed using the 3Shape software (3Shape, Copenhagen, Denmark) [2,5]. The finish lines of the two abutments were traced, then a full anatomic bridge design was chosen, as shown in Figure 4. The cement space was set up for both abutments: cement gap, 0.03 mm; extra cement gap, 0.06 mm; and finish line thickness, 1 mm [1,41,42]. Finally, the occlusal and proximal contacts of the FDP were adjusted, as shown in Figure 5. The design was saved, ready for the CAM step either by milling or 3D printing.

**Figure 6:**
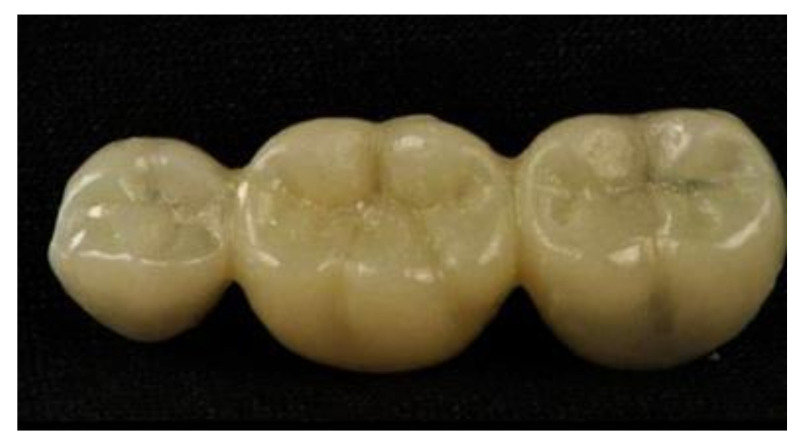
Milled provisional restorations.

The saved STL file was sent to the five-axis milling machine (CAM 5-S1 impression milling machine software, 3Shape, Copenhagen, Denmark) after positioning the FDPs in the desired position in the blank. The provisional material disc (The Tempo-CAD PMMA discs, on dent, Bornova, Turkey) was fixed to the machine holder. Then, the order was given to the mill to obtain the end product of the milled FDPs (n = 4). After milling, the supporting structures were removed, and the FDPs were finished and polished, as shown in Figure 6.

#### 2.6.2. 3D Printing the Tooth-Supported Provisional Dental Prosthesis (Group B)

The 3D Printing of four tooth-supported provisional dental prostheses (n = 4) was carried out using a Formlabs SLA 3D printer (Formlabs Inc., Somerville, MA, USA). The Formlabs temporary CB resin tank, resin cartridge, and build platform were inserted into the printer. A print job using Preform software was prepared by importing the saved design of the dental restoration STL file.

The files were oriented horizontally with the occlusal plane facing the build platform, and then supports were generated. The print job was then sent to the printer and the printing procedure was started. After the printing procedure, using the form wash unit, the FDPs were washed with clean IPA (≥99%). The form wash was set for 3 min.

Then, compressed air was used to dry the printed provisional restorations. The dried parts had a white, powdery coating on the print surfaces.

After drying, the printed FDPs were removed from the build platform by wedging the removal tool under the print raft and rotating the tool.

**Figure 7:**
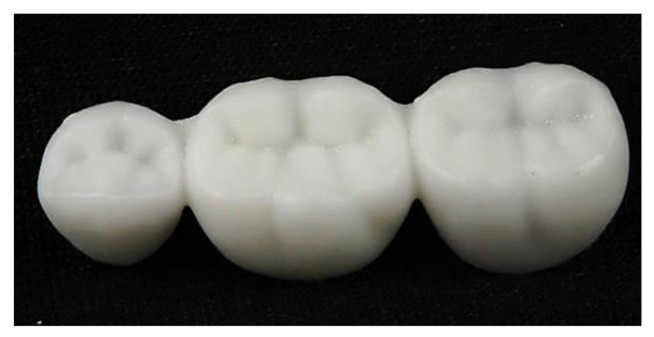
3D printed provisional restorations.

To maintain dimensional accuracy and biocompatibility, post-curing was carried out in two steps using the form cure unit as follows:

With the raft and supports still intact, the printed FDPs were placed in the form cure unit with the raft side down and cured at 60 °C (140 °F) for 20 min. Then, a handpiece with a cutting disc was used to separate between the supports and the raft. Next, sandblasting of the printed surfaces was performed to remove the white, powdery coating with a glass bead blasting material of 50 μm at a blasting pressure of 1.5 bar. To adjust the fit and contour of the FDPs, a finishing procedure was carried out using a dental handpiece and carbide rotary burs, as shown in Figure 7.

### 2.7. Measurement of 3D Accuracy of Test Working Models

#### 2.7.1. Digitizing the Models of the Two Test Groups

The four working models of the two test groups (milled and printed) were scanned one after the other by using a high accuracy (7 µm) desktop E3 scanner (3Shape, Copenhagen, Denmark) [43]. This was used to digitize the models of the two test groups, hence enabling the point cloud format of the model, CAD test model (CTM) data, which allows for a 3D analysis method of all point clouds compared to that of the reference model [8,24,44].

#### 2.7.2. STL Superimposition Procedures

**Figure 8:**
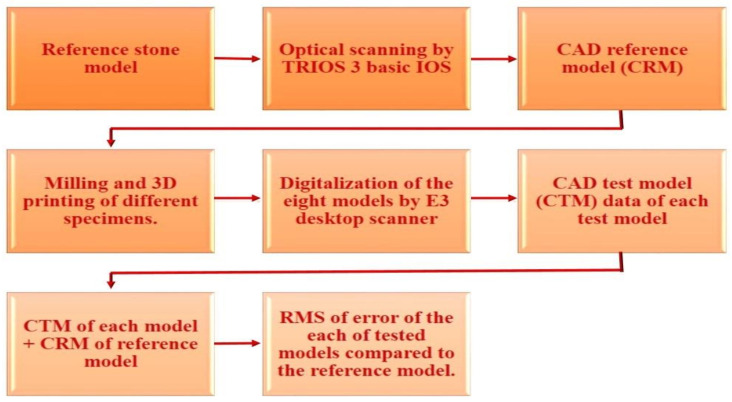
The process of 3D accuracy measurement by superimposition.

The STL dataset of each model from the test groups (CTMs) was superimposed to the STL dataset of the control reference model (CRM) using the Geomagic Control X superimposition software (Artec 3D, Santa Clara, CA, USA) to measure the accuracy of the test models in term of trueness, mainly as it is the main concern in assessing the ability of the milling machine and the 3D printer to provide a precise working model in comparison to the reference model, as shown in Figure 8.

**Figure 9:**
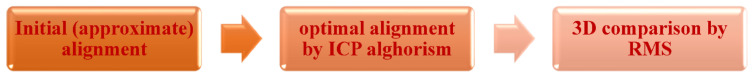
3D analysis with Geomagic software.

In the 3D analysis, the STL of reference model (CRM) and test models (CTMs) were first aligned, and a 3D comparison was then performed. The approximate positions of reference and test models were initially aligned in the alignment process, followed by optimal alignment. The iterative closest point (ICP) algorithm uses optimal alignment to minimize the difference between point clouds. Initial alignment allows for the approximate position to be aligned, and optimal alignment can be used to align and estimate the minimum distance of each corresponding point cloud of the STL files of the reference and test models as shown in Figure 9 [45,46].

After the alignment, the 3D comparison was performed. The distances between all corresponding points were calculated by using the RMS formula where mean distance between corresponding points was calculated, corresponding to the mean value of errors, by using the following formula:

(1) $$\mathrm{RMS}=\frac{1}{\sqrt{n}}\sqrt{\sum_{i=1}^{n}D_{i}^{2}}$$

*Di* represents the gap distance of the point (*i*) of reference and test models, and (*n*) is the number of all points evaluated of the point cloud of each test model. A higher calculated RMS value indicated a significant error, i.e., the difference in the attributes between reference and measurement data [46].

**Figure 10:**
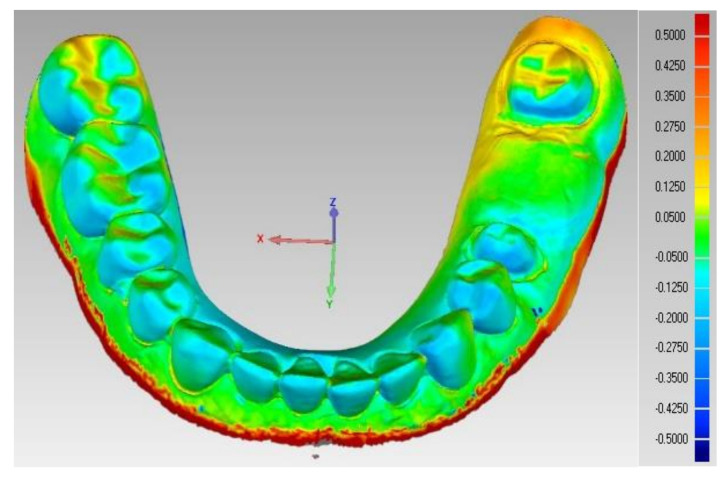
Color map representing the 3D accuracy of the test model compared to the control reference model.

A color difference map was set in a color range of 500 µm in the software program as shown in Figure 10. The red color region with positive error (+10 to +500 µm) indicates that the test model is located above the reference model, model expansion, and the blue color region (negative error: +10 to +500 µm) that the test model is located below the reference model, model shrinkage.

The color map corresponds to the RMS value, and it is a representative qualitative map for the 3D accuracy of the test model [46].

### 2.8. Measuring the Marginal Fit of the Two Test Groups

**Figure 11:**
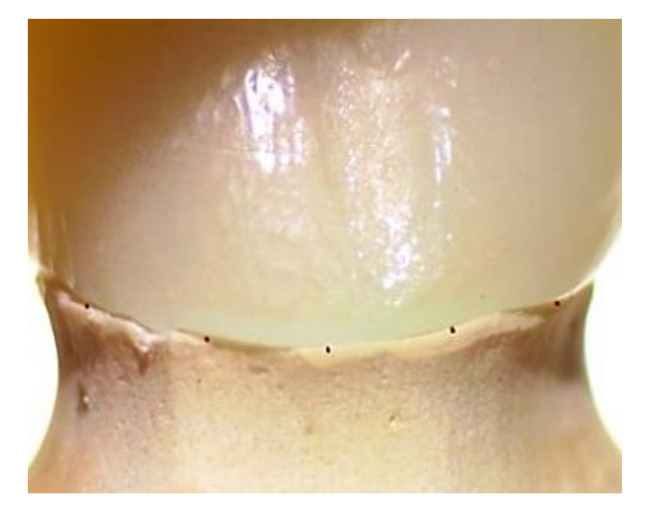
Equidistant points of measurements on stereomicroscope.

The vertical marginal gap distance for each FDP was measured using a stereomicroscope (Euromex, Microscope BV, Arnhem, The Netherlands). Images for the margins were captured with a specified camera in the microscope with 10× magnification. Five equidistant measurement points were taken from each surface (buccal, lingual, mesial, and distal) with a total of 20 points for each retainer of the FDP as shown in Figure 11 [47]. Measurements were recorded in microns, and the mean of 20 points was recorded for statistical analysis. Digital image analysis software (Image J 1.43U image analysis software) was used to measure and evaluate the gap. The measured parameters were expressed in pixels and converted to microns using this software. Standardization was carried out by comparing an object of known size (a ruler in this study) with a scale generated by the software.

### 2.9. Data Analysis

Data were checked for normality using the Shapiro–Wilk normality test. Marginal gap distance data showed non-normal (non-parametric) distribution, while accuracy data showed normal (parametric) distribution.

Data are presented as median, range, mean and standard deviation (SD) values. For non-parametric data, the Mann–Whitney U test was used to compare the two groups. For parametric data, Student’s *t*-test was used to compare the two groups. The significance level was set at *p* ≤ 0.05. Statistical analysis was performed with IBM SPSS Statistics for Windows, Version 23.0. Armonk, NY, USA: IBM Corp.

## 3. Results

### 3.1. Concerning the 3D Accuracy of the Working Model

**Figure 12:**
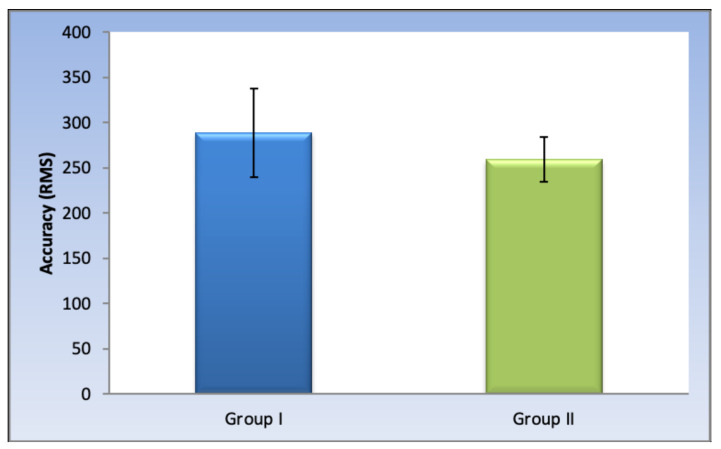
Bar chart representing mean and standard deviation values for the accuracy of the two groups.

In Table 1 and Figure 12, the results showed that there is no statistically significant difference between the accuracy of the milled and 3D printed groups (*p*-value = 0.321, Effect size = 0.764).

### 3.2. Three-Dimensional Accuracy Color Difference Map

The 3D color maps recorded the deviation patterns between the tested working models of both tested groups and the control reference model. Yellow and red marked the models’ expansion compared to the control model. The light to dark blue area represented the shrinkage of the tested model. The pattern of deviation was heterogeneous.

**Figure 13:**
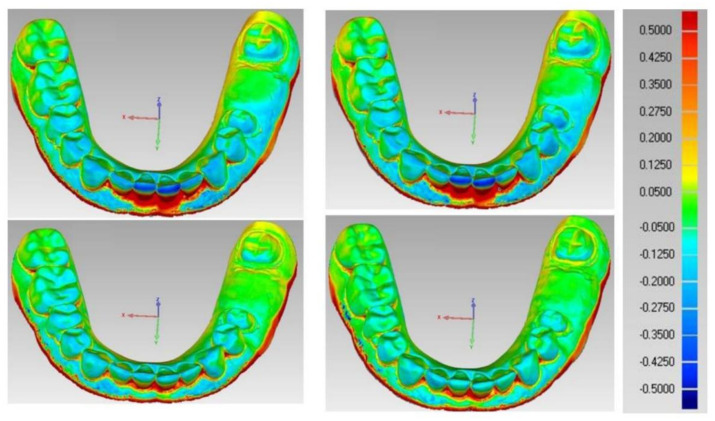
Color map of milled models.

Concerning the color difference map of the four milled working models, shown in Figure 13, yellow and red areas in the labial region represent model expansion. In contrast, light and dark blue areas in the incisal and occlusal regions represent model shrinkage.

**Figure 14:**
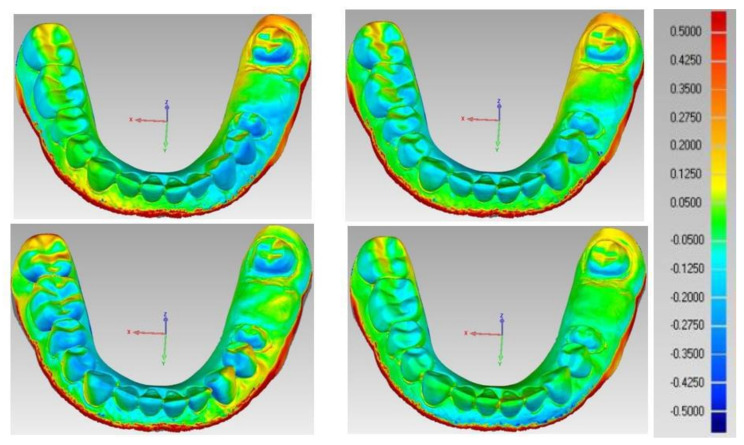
Color map of printed models.

However, in the color difference map of the four 3D printed working models shown in Figure 14, the yellow and red areas in the posterior occlusal region represent model expansion. In comparison, light and dark blue areas in the incisal anterior regions represent model shrinkage.

### 3.3. Concerning the Marginal Fit

**Figure 15:**
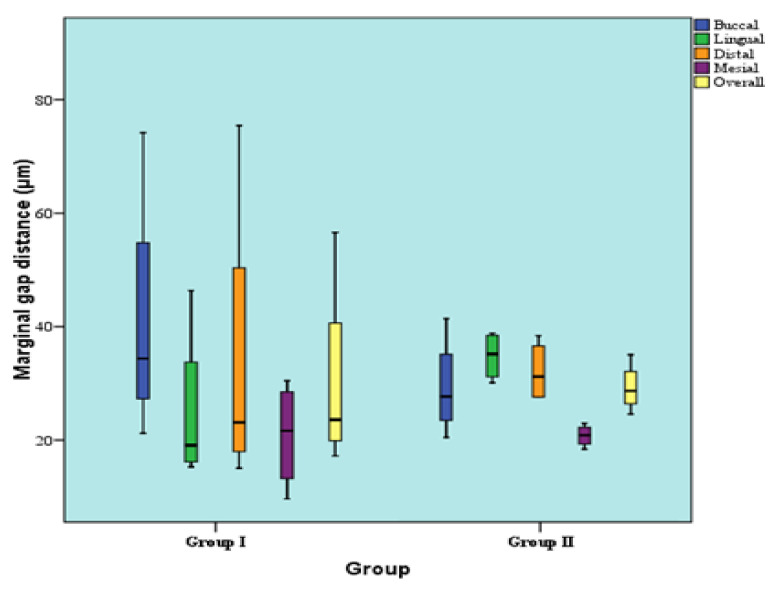
Box plot representing median and range values for the marginal gap distances in the two groups (distal retainer).

In Table 2 and Figure 15, regarding the distal retainer, at the buccal, lingual, distal, as well as mesial surfaces, there was no statistically significant difference between marginal gap distances of the two groups (*p*-value = 0.149, effect size = 1.187; *p*-value = 0.248, effect size = 0.894; *p*-value = 0.564, effect size = 0.417; and *p*-value = 0.386, effect size = 0.643). Regarding the overall gap distance regardless of surface, there was no statistically significant difference between the marginal gap distances of the two groups (*p*-value = 0.773, effect size = 0.205).

**Figure 16:**
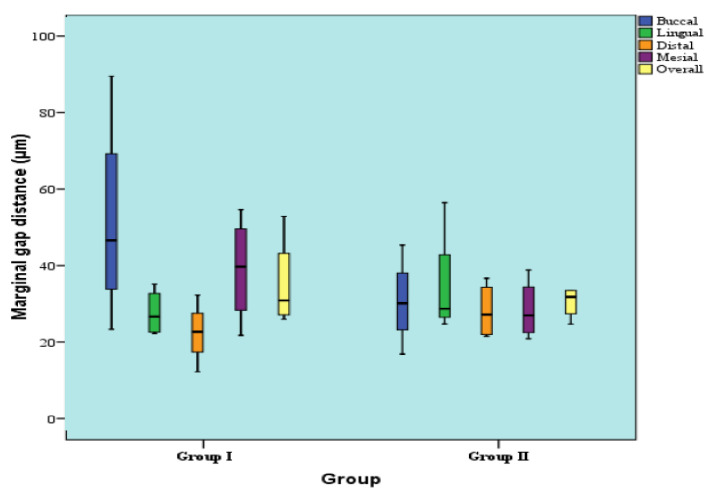
Box plot representing median and range values for marginal gap distances in two groups (mesial retainer).

For the mesial retainer, at the buccal, lingual, distal, as well as the mesial surfaces; there was no statistically significant difference between marginal gap distances of the two groups (*p*-value = 0.248, effect size = 0.894; *p*-value = 0.386, effect size = 0.643; *p*-value = 0.149, effect size = 1.187; and *p*-value = 0.386, effect size = 0.643). Regarding overall gap distance regardless of surface, there was also no statistically significant difference between marginal gap distances of the two groups (*p*-value = 0.773, Effect size = 0.205) as shown in Table 3 and Figure 16.

**Figure 17:**
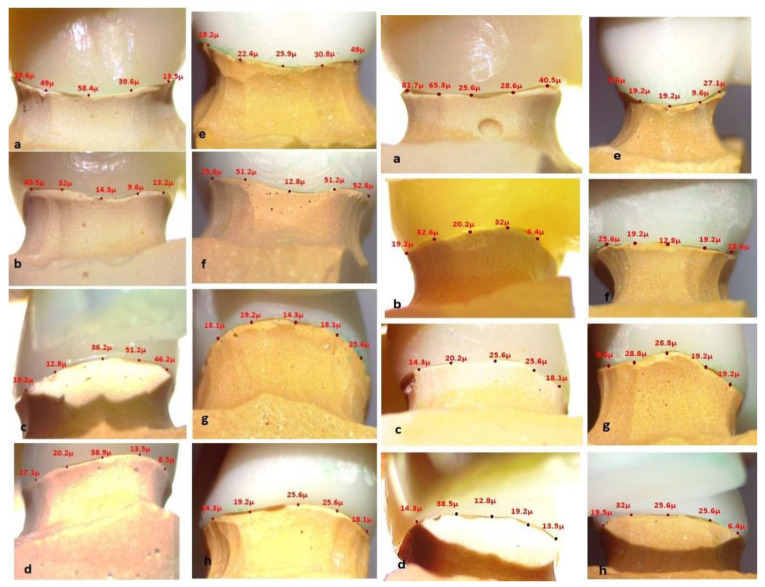
3D printed provisional restoration (molar and premolar) at different surfaces: (**a**–**d**) milled and (**e**–**h**) 3D printed (buccal, lingual, mesial, and distal).

Figure 17 demonstrates representative stereomicroscopic images of both the mesial and distal retainers showing the difference in marginal fit of both milled and 3D printed groups.

## 4. Discussion

Digital fabrication techniques have gained popularity recently and have efficiently substituted for conventional ones. These techniques allow for the fabrication of restorations with virtual models and dies using the CAD software without the need for a working model, along with digital manufacturing (CAM), whether subtractive (milling) or additive (3D printing) techniques [9]. However, in some cases, a working model is still essential during the digital workflow [8,39].

Likewise, provisional restorations play an indispensable critical role in the success of the treatment plan of complex cases [48]. Long-term provisionalization of these cases can be adequately achieved by using the CAD/CAM fabrication techniques that offer restorations of better quality [48,49].

The three-dimensional accuracy of the milled and 3D printed working models was assessed using specialized 3D analysis software. Because the 3D analysis method can analyze the accuracy of all point clouds of a virtual cast in the X, Y, and Z axes, it can detect the errors found in the whole tested volume of the fabricated working model [46]. This offers a more accurate assessment compared to the previously adopted linear measurement methods that only determine limited reference points and compare the distance between them.

Geomagic Control X software was used as it is one of the most common and precise 3D analysis software available. It also allows for 3D comparison, unlike other software that utilizes surface or mesh distance comparisons. It also allows for modifying the color map extents and customizing the range according to the object being measured and its clinical significance, unlike materialize 3-Matic color map software, as an example, that does not possess this feature [46].

The vertical marginal gap was assessed by using a stereomicroscope, which is a non-destructive measurement method, according to Romeo et al. [50]. In addition, Yucel et al. [51] stated that the direct imaging technique using a microscope with image analysis software permits non-destructive multiple measurements.

In the current study, a total of 20 reference points were measured for each retainer of the FDP to cover the margin circumferentially. It was supported by a study conducted by Groten et al. [52].

They claimed that a range of 20 to 25 measurements per crown is sufficient to accurately evaluate close to 50 measurements per crown (optimum number). Therefore, this study was able to obtain adequate information about the gap size and assured a statistical accuracy of the results.

The first and the second null hypotheses of the present study were accepted as there was no significant difference in the three-dimensional accuracy of working models fabricated with two digital methods (three-dimensional printing and milling). In addition, no significant difference was observed in the vertical marginal fit between three-dimensionally printed and CAD/CAM milled tooth-supported provisional dental prosthesis.

According to the literature review, there is currently no consensus on the clinically acceptable value for working model accuracy, and hence, different studies mentioned variable values as a reference to the clinical significance of their studies. However, a systemic review published by Etemad-Shahidi et al. [21] of a total of 28 studies mentioned that most of the studies [53,54,55,56,57,58,59,60,61,62,63,64] defined the clinically acceptable error to be less than 500 µm. Consequently, the 3D accuracy values of the milled and printed working models in the present study are within this range (the mean RMS of error for the milled and printed working models was 288.8 and 259.1 µm, respectively).

The present study results showed no statistically significant difference in the 3D accuracy between milled and 3D printed working models. Despite being statistically insignificant, the findings of this in vitro study showed that the 3D printed models showed fewer 3D deviations than the milled ones. This result was in accordance with the more recent studies [8,26] that credited this result to the fact that the smallest bur used in the milling process was 1 mm. This limited the accurate reproduction of areas that were less than 1 mm. A bur of less than 1 mm could not be used on milling PMMA resin as it is quickly heated. This would cause the resin to melt and interlock in the flutes of the bur causing fracturing of the bur during the fabrication process [8,28].

The color difference maps showed heterogeneous 3D deviations in milled and 3D printed models. However, the dominant 3D deviation found in the milled models was the model expansion, where the color maps of the milled models showed red and yellow areas in the lower incisors region labially and interdentally. These areas have the smallest radiuses that require a smaller milling bur for accurate geometry reproduction. Similar results were encountered by Marcel et al. [28]. On the other hand, model shrinkage was more dominant in 3D printed models, which was seen as scattered light and dark blue colors in the map. This result was in agreement with Choi et al. [26] who attributed this phenomenon to the post-curing procedure that may result in some shrinkage that the addition of any successive layers will not compensate.

The result of this study was in disagreement with earlier studies conducted by Yau et al. [25]. They found that milled models are more accurate with a better surface finish than 3D printed models. The decreased accuracy of 3D printed models was attributed to the stepped surfaces formed by successive layering and model deformation resulting from polymerization shrinkage [8,26].

The result of the current study also disagreed with a comparative study performed by Jeong et al. [8]. They found that the RMS value of the model manufactured by the milling method was significantly higher than the RMS value of the model produced by the 3D printing method. The variation in the result was due to the difference in the research methodology, as the authors tested the accuracy of half arch models, while in the present study, the accuracy was tested for the full arches. Son et al. [46] clarified that the differences in measuring the 3D accuracy become more significant as the volume to be measured becomes smaller.

During measurement of the vertical marginal fit in the present study, the dies were positioned in a holding jig machine to hold the tested FDP on the die with a standardized force for all the specimens for accurate, standardized measurements. Marginal gap measurement was carried out without cementation to exclude the effect of cementation technique variations [65].

The vertical marginal gap values of the milled and 3D printed FDPs were within the clinical acceptance range as per McLean et al. [66], which is less than 120 µm. The present study results showed that the 3D printed FDPs had statistically insignificant but better marginal adaptation and less marginal discrepancy. The insignificant difference may be attributed to the high precision and the continuous innovations in the CAM, whether for milling or 3D printing. At the same time, the better vertical marginal fit of the 3D printed group over the milled one could be attributed to the fact that the smallest bur used in the milling process was 1 mm. This limited the accurate reproduction of areas less than 1 mm [8,26].

This result was in agreement with the studies performed by Haddadi et al. [67], who found that there is no statistically significant difference in the value of the vertical marginal gap between the provisional crowns fabricated by milling and those fabricated by 3D printing. They concluded that 3D printing could effectively replace milling in the fabrication of provisional restorations.

On the other hand, several studies [1,5,68,69,70] concluded that 3D printing offers better marginal adaptation compared to milling. Park et al. [68] and Lee et al. [5] attributed the decreased marginal fit of milled-implant-supported restoration to the milling bur diameter. They reported that the curved parts of the provisional restoration margin were more precisely fabricated by 3D printing than milling due to the limitations of motion of the milling machine axes and bur diameter. Moreover, Elfar et al. [70] concluded that the higher accuracy of the 3D printing was attributed to the incremental layering process during fabrication that allows for accurate reproduction of all details, adequate compensation of the polymerization shrinkage, and better marginal fit compared to milling. Lastly, Alharabi et al. [1] credited the higher vertical marginal gap of the milled restorations to the tolerance of the milling burs and their wear. They claimed that any surface detail less than the diameter of the milling bur would be over-milled and lead to loose and inaccurate restoration.

Contradicting results were reported by Savencu et al. [71]. They found that the best vertical marginal gap values were obtained for the milled metal copings, followed by the 3D printed ones. They declared that the decreased accuracy of the 3D printed copings is due to the accumulation of errors at different stages of fabrication, the design segmentation by the printing software, processing, and during the printing process itself. The shrinkage during building and post-curing led to a more considerable marginal discrepancy.

The limitations of this study include failing to reproduce clinical situations fully, namely saliva, patient movement, and anatomical features (tongue, lips, and cheeks) during scanning and designing. In addition, the scanner used during the digitalization of the tested models was not an industrial “reference scanner”. The present study was limited to analyzing the fit of three-unit FDPs. Moreover, the present study investigated the accuracy of SLA 3D printing technology only. Finally, the provisional restorations were not tested after aging and thermocycling.

Therefore, further research should be conducted with simulated oral environmental and clinical factors. Moreover, investigating the fit of long-span FDPs and oral rehabilitation cases is recommended. Finally, the upcoming studies should test 3D printing technologies other than the tested SLA technology.

The final outcome of this study is that additive 3D printing technology can replace its subtractive counterpart during the construction of provisional restorations for its maximum accuracy, precise fit, and cost-effectiveness using the digital workflow.

## 5. Conclusions

Within the limitations of the present study, the following points could be concluded:

1. The working models manufactured by the 3D printing and milling techniques showed comparable accuracy.
2. The vertical marginal gap values of provisional restorations fabricated by the two tested manufacturing methods (milling and 3D printing) were within the acceptable clinical range of 120 microns.
3. The 3D printed provisional restorations showed comparable marginal fit to the milled ones.

## Figures and Tables

**Table 1:** Descriptive statistics and results of Student’s *t*-test for comparison between accuracy (RMS) of the two groups.

Group I (n = 4)		Group II (n = 4)		*p*-Value	Effect Size (d)
Mean	SD	Mean	SD		
288.8	49	259.1	24.8	0.321	0.764

**Table 2:** Descriptive statistics and results of Mann–Whitney U test for comparison between marginal gap distances (µm) in the two groups (distal retainer).

Surface	Group I (n = 4)	Group II (n = 4)	*p*-Value	Effect Size (d)
Buccal				
Median (Range)	41.1 (27.9–80.9)	28.3 (21.2–42)	0.149	1.187
Mean (SD)	47.7 (23)	30 (8.8)		
Lingual				
Median (Range)	25.7 (22–53)	35.8 (30.8–39.5)	0.248	0.894
Mean (SD)	31.6 (14.5)	35.5 (4.3)		
Distal				
Median (Range)	29.8 (21.7–82.1)	31.9 (28.2–39.1)	0.564	0.417
Mean (SD)	40.8 (27.8)	32.7 (5.4)		
Mesial				
Median (Range)	28.3 (16.3–37.1)	21.5 (19.1–23.6)	0.386	0.643
Mean (SD)	27.5 (9.4)	21.4 (1.9)		
Overall				
Median (Range)	30.3 (23.9–63.3)	29.3 (25.2–35.7)	0.773	0.205
Mean (SD)	36.9 (17.9)	29.9 (4.3)		

**Table 3:** Descriptive statistics and results of Mann–Whitney U test for comparison between marginal gap distances (µm) in the two groups (mesial retainer).

Surface	Group I (n = 4)	Group II (n = 4)	*p*-Value	Effect Size (d)
Buccal				
Median (Range)	45.9 (22.9–89.3)	30.8 (17–46.5)	0.248	0.894
Mean (SD)	50.9 (27.9)	31.3 (12.1)		
Lingual				
Median (Range)	25.8 (21.3–34.4)	29.3 (25.1–58)	0.386	0.643
Mean (SD)	26.8 (6.2)	35.4 (15.2)		
Distal				
Median (Range)	21.8 (11.2–31.5)	27.7 (21.8–37.5)	0.149	1.187
Mean (SD)	21.5 (8.3)	28.7 (7.7)		
Mesial				
Median (Range)	39 (20.8–54)	27.5 (21.2–39.8)	0.386	0.643
Mean (SD)	38.2 (14.2)	29 (8.2)		
Overall				
Median (Range)	30 (25.1–52.3)	32.5 (25.1–34.3)	0.773	0.205
Mean (SD)	34.4 (12.4)	31.1 (4.3)		

## Data Availability

The data presented in this study are available on request from the corresponding author.
